# *“It’s burning down there”:* A Mixed-Methods Study on Sexually Transmitted and Other Vaginal Infections Among Adolescent Girls and Young Women in South Africa

**DOI:** 10.1080/19317611.2026.2637641

**Published:** 2026-03-12

**Authors:** Zoe Duby, Kate Bergh, Kim Jonas, Mari Lotvonen, Caroline Kuo, Catherine Mathews

**Affiliations:** a Health Systems Research Unit, South African Medical Research Council, Cape Town, South Africa; b School of Public Health, University of Cape Town, Cape Town, South Africa; c Networking HIV/AIDS Community of Southern Africa (NACOSA), Cape Town, South Africa; d American University, Washington, DC

**Keywords:** Sexually transmitted infections (STIs), adolescent girls and young women, South Africa, stigma, shame

## Abstract

**Objectives:**

South Africa has one of the highest rates of sexually transmitted infections (STIs) globally, with adolescent girls and young women (AGYW) disproportionately affected due to intersecting biological, behavioral, structural, and social determinants. While HIV prevention receives sustained attention, other STIs remain under-addressed despite high prevalence and increased HIV transmission risk. This study explored STI- and vaginal infection-related knowledge, attitudes, experiences, and health-seeking behaviors among South African AGYW.

**Methods:**

A convergent parallel mixed-methods approach integrated a household survey of 4932 AGYW aged 15–24 years with 68 qualitative interviews. Descriptive quantitative analyses and qualitative thematic analysis were performed.

**Results:**

Only 16.1% (794/4932) of surveyed AGYW reported an STI diagnosis by a medical professional. Genital itching was the most common self-reported symptom (17.5%), but a further 22.5% of participants preferred not to disclose symptoms experienced in the past year. Among those reporting STI-related symptoms, most sought care from clinics/hospitals (57.1%), with older participants more likely to seek treatment. Qualitative findings revealed variation in STI knowledge, from complete unawareness to detailed understanding of transmission and symptoms, although inaccuracies persisted. Whilst school was the most common source of STI information, peers served as important information sources, albeit often inaccurate. Family members, particularly older women, were also sources of STI education, although discussions were limited by sexuality communication restrictions. Most participants had never discussed vaginal infections or STIs with anyone, illustrating communication gaps.

**Conclusions:**

In examining knowledge, attitudes, experiences and health seeking behaviors of South African AGYW in relation to vaginal infections and STIs, our study found that persistent shame, stigma, and embarrassment act as key barriers to enacting preventative and health-seeking behaviors that could treat and prevent further infections. Findings underscore the need for comprehensive, accurate STI and vaginal health education and service provision for AGYW that addresses misconceptions, stigma and shame.

## Introduction

Sexually transmitted infections (STIs) other than HIV – such as human papillomavirus (HPV), chlamydia, gonorrhea, syphilis – have been referred to as a hidden epidemic in sub-Saharan Africa generally, and South Africa more specifically (Dookhith, 2022). South Africa faces one of the world’s highest burdens of STIs, with the highest reporting globally for STI-related disability-adjusted life years (Deng et al., 2025). The prevalence rates of treatable STIs among South African women are very high, with some estimates suggesting that one in four women in South Africa is infected with at least one bacterial STI (Dookhith, 2022).

Similarly to HIV, adolescent girls and young women (AGYW) aged 15–25 years bear a disproportionate share of South Africa’s STI burden, with the highest STI incidence among young women in the age group 20–24 years specifically (Frank et al., 2023; Harryparsad et al., 2023; Price et al., 2024). Available evidence clearly demonstrates that the cumulative burden of curable STIs – chlamydia, gonorrhea, syphilis, and trichomoniasis – among AGYW is substantial. Gonorrhea prevalence among AGYW shows considerable variation by geographic location and study population, with prevalence rates of up to 11% (Jarolimova et al., 2023). Chlamydia prevalence has shown to be substantially higher among AGYW than older women, with prevalence rates estimated as high as 42% in some communities, approximately four times as high as older cohorts of women (Harryparsad et al., 2023; Jarolimova et al., 2023). Human papillomavirus (HPV) infection, which alongside HIV and herpes simplex, is among the incurable STIs, shows extremely high prevalence rates, ranging from 56.5% to 76.1% among AGYW aged 15–25 years (Travill et al., 2024). This is despite the fact that the South African state provides the HPV vaccine to AGYW through the Department of Health’s national school-based vaccination programme.

Vaginal infections such as thrush (candidiasis) and bacterial vaginosis (BV) are not classified as STIs but can be transmitted through sexual contact and are associated with an increased risk of HIV (Happel et al., 2022). The symptoms of thrush and BV are similar to many STIs which can cause confusion and inappropriate treatment responses among women experiencing symptoms (Bilardi et al., 2016). Evidence suggests prevalence of BV among South African AGYW as high as 44% (Mullick et al., 2023).

The sustained high prevalence rates of HPV, syphilis, gonorrhea, and BV among AGYW in South Africa present significant public health challenges due to the long-term consequences of untreated infections, including increased HIV susceptibility and transmission risks, reproductive health complications, and cervical cancer risk (Barnabas et al., 2017; Dadzie et al., 2022; Harryparsad et al., 2023; Kaida et al., 2018). In particular, the high prevalence of STIs among AGYW increases HIV transmission risk, driving HIV incidence in this population, highlighting a critical need for improved integrated and comprehensive sexual and reproductive health (SRH) services (Francis et al., 2018).

Despite the high prevalence of STIs and vaginal infections, evidence suggests substantial knowledge gaps relating to STIs among South African AGYW (Ditshwane et al., 2023). In addition to knowledge gaps around symptom recognition, research among young people in South Africa indicates numerous barriers to seeking timely healthcare for STIs including treatment of curable STIs and infections (Ditshwane et al., 2023; Mokgatle et al., 2021). This is concerning as delaying treatment, or leaving STIs untreated, can have serious long-term implications (Delany-Moretlwe et al., 2023).

Due to the focus on HIV – a pressing issue and a priority for funding – STIs among AGYW in South Africa have been somewhat neglected as a research topic. However, this is a missed opportunity for integrated HIV-STI care. For example, evidence suggests that STI rates are concerningly high among individuals using pre-exposure prophyliaxis (PrEP) for prevention of HIV, with studies showing that more than a quarter of PrEP users tested positive for an STI (Joseph Davey et al., 2025; Mullick et al., 2023). Given this context, there is a need to understand the levels of knowledge around STIs, and the barriers to AGYW in South Africa seeking care and treatment for STIs. Doing so can guide efforts to cure treatable STIs and vaginal infections, to provide appropriate care for untreatable STIs including putting in place prevention measures to limit onwards transmission, and maximize HIV prevention and treatment as an opportunity to also address the high prevalence of vaginal infection and STIs among AGYW. In this mixed-methods study we sought to shed light on the knowledge, attitudes, experiences and health seeking behaviors of South African AGYW in relation to vaginal infections and STIs.

## Methods

This study employed a convergent parallel mixed-methods approach combining a quantitative survey with qualitative in-depth interviews.

### Study sample and setting

The study sample included AGYW aged 15–24 years from 24 sub-districts across eight provinces in South Africa: KwaZulu-Natal, Mpumalanga, Eastern Cape, Free State, Limpopo, Gauteng, North West and Western Cape. Prior to recruitment, political and traditional leadership buy-in was obtained, to ensure that access to the study communities had been negotiated. In some communities, most notably in Limpopo, all research activities were suspended following incidents in which the safety of the research team was compromised, thus preventing achievement of sample size targets.

### Data collection procedures

#### Quantitative methods: Household survey

The quantitative component comprised a representative household survey conducted from February to May 2024 in the sampled 24 sub-districts. Two sites were purposefully sampled within each sub-district, generating 48 sites across eight provinces. At each study site, 100 AGYW were recruited to take part in the survey, equating to an expected sample size of 4800. To be eligible for participation in the survey, the following criteria had to be met: female resident of selected household aged 15–24 years, willing and able to provide written informed consent or parental consent for those under 18 years of age, willing to participate in the study and take part in study procedures.

In order to recruit participants, study staff approached households included in the sample, made appropriate introductions, explained the objectives of the study and the confidentiality of the household and individual information, and identified the head of household. All individuals in the household who met the eligibility criteria and were available were invited to participate in the study. Those who agreed to participate were asked to identify a location either inside or outside their home where the remainder of activities could be conducted with as much privacy and confidentiality as possible. Trained field staff first collected the dried blood spot (DBS) specimens from each participant to test for HIV using a finger prick. Then the participant was shown how to self-administer the audio-assisted electronic questionnaire using a handheld tablet provided by the study. Apart from the first questionnaire items capturing geographical information and the unique barcode, the participant self-completed all questions with the option of listening to each question in English or in their choice of 10 official South African languages that were spoken in the study areas.

Questions pertaining to STIs (see Supplementary Information) were developed by researchers in the Health Systems Research Unit at the South African Medical Research Council for the baseline survey evaluation of the My Journey Programme. These questions asked participants about STI diagnoses from healthcare workers, STI symptoms experienced in the past year and where treatment for these symptoms was obtained. Given prevailing low levels of knowledge about STIs, this allowed the researchers to obtain information about participants’ exposure to STIs without asking participants about the specific STIs they may have had.

### Qualitative methods

The qualitative sample was drawn from the household survey described above. Survey participants were invited to participate in additional telephonic in-depth interviews which took place in the period March to May 2024. Those willing provided their cell phone numbers and later were contacted by an interviewer. Interviewers contacted participants on the cell phone numbers providers, and conducted semi-structured in-depth interviews via Microsoft Teams software calling function, using the platform’s built‑in recording function to audio record interviews, with informed consent obtained prior to recording. As shown in Table 1, interviews were conducted with a total of 68 AGYW aged 15–24 years (thirteen AGYW aged 15–17 years, and 55 AGYW aged 18–24). Interviews took place in participants’ choice of language (isiZulu, Sesotho, Setswana, Afrikaans, isiXhosa or English), at a time convenient for the participant, following a semi-structured interview guide (see Supplementary Information), and lasted between 20–120 minutes. Female researchers fluent in the study languages, with tertiary qualifications ranging from Bachelors to Masters degrees and trained on qualitative interview techniques and research ethics conducted the interviews. The researchers did not have any prior or existing relationships with study participants. Prior to conducting the interviews, interviewers checked with participants if they were in a safe, private and comfortable place to continue with the interview. Interviews were audio recorded with participants’ consent, labeled with unique codes, and saved in a secure, password protected online drive.

## Data analysis

Following a convergent parallel mixed methods process, the quantitative and qualitative datasets were analyzed independently in parallel, then merged during interpretation.

### Quantitative data analysis

Descriptive analyses were conducted in Stata (Stata SE 18.0, StataCorp, Texas, USA). Descriptive statistics, stratified by age group, were used to report the frequencies, percentages, and 95% confidence intervals (CIs) for survey responses pertaining to STIs from the survey data. For the 95% CIs, we accounted for the survey design by stratifying by sub-district and clustering at the household level.

### Qualitative data analysis

English transcripts were produced from interview audio recordings, either through direct transcription of English audios, or direct translation of audios in other languages. Transcripts were anonymized to protect participant confidentiality. A Collaborative Qualitative Analysis process grounded in thematic analysis was used, in which English transcripts were analyzed collaboratively by three analysts using shared password protected online summary memo and coding documents, which were continually updated to capture transcript coding, alongside emerging themes and analysts’ reflections on the data (Birks et al., 2008; Richards & Hemphill, 2018). Analysis followed an integrated and cyclical process using a set of preliminary deductive codes based on three main thematic areas covered in the interview guide: STI knowledge, STI experiences, STI communication. These key codes were built upon through inductive development and refinement following an iterative process of reading the textual data and identifying emergent themes, combined with dialogue and consensus (Milford et al., 2017).

## Ethical considerations

Ethical approval for the study was granted by the Human Ethics Research Committee at the South African Medical Research Council (EC027-8/2023). All participants provided informed consent. For participants under 18 years of age, parental/guardian consent was obtained. Participants received R200 (approximately US $11) reimbursement for their participation. Referral systems were in place for any participants requiring mental health support or social protection.

## Findings

### Survey findings

Across study arms, 37,714 dwellings were visited, 22,263 households were screened, 5150 AGYW were invited and 5,025 participated (97.5% response rate). Of the 5,025 survey participants, 4932 (98.2%) had available DBS test data and were included in our analysis (Table 1). Table 2 describes participants’ experiences of STIs, stratified by age group. The percentage of participants in the <18 years age group was 34.7%, compared to 65.3% in the ≥18 year age group (min = 15 years, max = 24 years, mean = 19 years).

**Table 1.** Study samples by province.

Site code	Province	Survey sample (N)	Qualitative sample (N)
KZN	KwaZulu-Natal	843	7
MPU	Mpumalanga	857	14
EC	Eastern Cape	826	3
FS	Free State	872	11
LM	Limpopo	288	0
GAU	Gauteng	483	8
NW	North West	318	10
WC	Western Cape	445	15
	Totals	4932	68

**Table 2.** Participants’ self-reported diagnosis of sexually transmitted infections and STIs symptoms among all participants (n = 4932).

Variable	<18 years		≥18 years		Total	
	Freq (%)	95% CI	Freq (%)	95% CI	Freq (%)	95% CI
Participant has ever been diagnosed with an STI by a doctor or nurse	161 (9.4)	(8.3–10.6)	633 (19.7)	(18.2–21.2)	794 (16.1)	(15.1–17.2)
Possible STI symptoms experienced by participants on their penis – vagina – anus/bum in the past year						
Itching	217 (12.7)	(11.1–14.4)	646 (20.1)	(18.8–21.4)	863 (17.5)	(16.4–18.6)
Lumps	53 (3.1)	(2.4–4.0)	116 (3.6)	(3.1–4.2)	169 (3.4)	(3.0–3.9)
Warts or a rash	79 (4.6)	(4.0–5.4)	181 (5.6)	(5.0–6.3)	260 (5.3)	(4.9–5.7)
Redness	38 (2.2)	(1.6–3.1)	114 (3.5)	(3.0–4.1)	152 (3.1)	(2.7–3.5)
Unusual smell from yellow or brown or white discharge	91 (5.3)	(4.4–6.3)	314 (9.8)	(8.8–10.8)	405 (8.2)	(7.5–9.0)
Pain or burning feeling when urinating or having sex	74 (4.3)	(3.3–5.7)	269 (8.4)	(7.4–9.4)	343 (7)	(6.1–7.9)
I did not experience any of these on my private parts	899 (52.5)	(49.5–55.4)	1476 (45.9)	(43.7–48.0)	2375 (48.2)	(46.2–50.1)
Prefer not to answer	411 (24)	(21.2–27.1)	700 (21.7)	(19.9–23.7)	1111 (22.5)	(20.7–24.4)

**Table 3.** Places where participants received treatment for a sexually transmitted disease (STI) among participants who reported having a symptom of an STI in the past year (n = 1446).

Variable	<18 years		≥18 years		Total	
	Freq (%)	95% CI	Freq (%)	95% CI	Freq (%)	95% CI
Clinic or hospital	141 (35)	(30.0–40.3)	685 (65.7)	(63.1–68.1)	826 (57.1)	(55.1–59.2)
Mobile clinic	43 (10.7)	(7.1–15.7)	118 (11.3)	(8.9–14.3)	161 (11.1)	(9.0–13.7)
Doctor	40 (9.9)	(7.2–13.6)	100 (9.6)	(7.6–12.1)	140 (9.7)	(8.2–11.4)
School nurse	17 (4.2)	(2.6–6.7)	21 (2)	(1.4–2.9)	38 (2.6)	(1.9–3.6)
Pharmacy	25 (6.2)	(4.5–8.4)	82 (7.9)	(6.5–9.5)	107 (7.4)	(6.2–8.8)
Traditional healer	8 (2)	(1.1–3.6)	15 (1.4)	(0.8–2.5)	23 (1.6)	(1.0–2.4)
Other	11 (2.7)	(1.4–5.1)	19 (1.8)	(1.2–2.7)	30 (2.1)	(1.5–2.8)
I did not get treatment	111 (27.5)	(22.9–32.7)	113 (10.8)	(9.3–12.6)	224 (15.5)	(13.7–17.5)
I did not experience any of these on my private parts	25 (6.2)	(4.5–8.5)	24 (2.3)	(1.4–3.6)	49 (3.4)	(2.6–4.4)
Prefer not to answer	24 (6)	(3.9–9.0)	30 (2.9)	(2.1–4.0)	54 (3.7)	(2.8–4.9)

In total, only 16.1% of all survey participants reported that they had ever been diagnosed with an STI by a medical professional, although this was significantly higher in the 18 and older age group (19.7%) compared to the under 18 age group (9.4%). In the survey, participants were asked about the following symptoms: itching, lumps, warts, rash, redness, unusual smell from yellow or brown or white discharge, and pain or burning feeling when urinating or having sex.

The most common symptom self-reported by participants was genital itching (17.5% across all age groups), with higher reporting (20.1%) among those aged 18 years and above, as compared to (12.7%) among those aged under 18 years. Other commonly reported symptoms included unusual smell from yellow or brown or white discharge (8.2%), pain or burning feeling when urinating or having sex (7.0%), warts or rash (5.3%), lumps (3.4%), and redness (3.1%). Notably, almost a quarter of survey respondents (22.5%) preferred not to answer the question about STI symptoms experienced in the past year.

As shown in Table 3, of those participants (n = 1446) who had reported an STI related symptom in the past year, the majority had sought treatment from a clinic or hospital (57.1%) across all age groups, with higher reporting amongst those aged 18 years and above (65.7%) as compared to those aged under 18 years (35.0%). Other sources of treatment in order of popularity included mobile clinics (11.1%), private doctors (9.7%), pharmacies (7.4%), school nurses (2.6%), and traditional healers (1.6%). Among those participants who had reported an STI related symptom in the past year, a total of 15.5% said that they did not get any treatment, with higher proportions of those AGYW under 18 years (27.5%) saying they did not get any treatment for their STI symptoms, as compared to the older age group (10.8%).

### Qualitative findings

#### AGYW STI knowledge

Most qualitative respondents stated that they had never had an STI themselves or known anyone else who had experienced an STI. As shown in Table 4, in terms of respondents’ knowledge about STIs in the qualitative data, levels of knowledge varied widely, with some respondents reporting no knowledge at all, some knew basics but not implications, and others articulated some degree of knowledge. Notably only a few AGYW aged 18 years and above had accurate STI knowledge, identifying behaviors which increase the chance of STI transmission including having condomless sex and multiple sexual partners. Whilst participants stated that STIs were transmitted through ‘sexual intercourse’, it was not specified whether this could be penile-vaginal and/or penile-anal, and none of the participants mentioned oral sex as a possible STI transmission vector.

**Table 4.** Illustrative quotations on Theme 1 – AGYW STI knowledge.

AGYW STI knowledge	
No knowledge	“(STIs)… I don’t know anything about them” (EC, 15–17)
Some limited knowledge	“I only know how you get them but I do not know their impact on the body system” (MPU, 18–24)
Knowledge of transmission	“STIs… are diseases you get from sexual intercourse” (EC, 18–24) “It is an infection you get sexually” (MPU, 18–24) “I know that they are caused by being sexually active without protection with multiple partners” (GAU, 18–24) “It is a vaginal infection you get when having unprotected sex with someone with the infection” (MPU, 18–24) “I learnt the cause of STIs… you get (them) when sleeping with multiple partners” (MPU, 18–24)
Knowledge of symptoms	“I know that if you have STIs your private parts they get… sores or stuff like that” (WC, 18–24) “I know that they are infectious if maybe you notice the symptoms like having discharge” (KZN, 18–24) “It itches on the vagina, there is also a smelling discharge that comes out (MPU, 18–24) “…infection, discharge being brown” (WC, 18–24) “I cannot tell when it can be spotted amongst women… ulcers are said to appear” (MPU, 18–24)
Knowledge of causes	“bacteria builds up and causes them… genital warts… herpes… that’s what I know about them” (GAU, 18–24)
Confusion about STIs/vaginal infections and symptoms	“There is one that is called yeast infection… you can get a smelly discharge with unfamiliar colour… you have brown or green colour with some itchiness underneath and you grow… warts, those are the symptoms of yeast infection” (EC, 18–24) “I know about herpes, when you have thrush on your genitals which starts off small itchy irritating… another sexually transmitted disease is HIV” (GAU, 18–24)
Inaccurate information/misconceptions	“It is a disease… you can even get it by using public toilets” (MPU, 18–24) “STI are found in your vagina and you get them if you sleep around and do not take a bath and you can infect all the boys that you sleep with” (MPU, 18–24) “(My friend) had it (an STI) it was obviously shocking, I mean we are friends, even going to the toilets, we share the same toilet. So, if it has reached this level on her, this could also harm (infect) me” (NW, 18–24)

Amongst those respondents with some knowledge of vaginal infections and STIs, some were able to describe symptoms such as discharge, malodor, genital sores, and itchiness, and some described symptoms related to yeast infections (thrush/candida). In addition to discharge, which was the most commonly recognized symptom by respondents, itchiness and malodor were also listed. One participant was also aware that some STIs are asymptomatic in females, but did not have comprehensive information about which ones. There was evidence of respondents confusing symptoms across different vaginal infections and STIs. Some of the AGYW knew terms for STIs and symptoms but mixed up symptoms matching them with the incorrect STIs or infections. In terms of knowledge of causes of STIs, respondents also appeared to confuse bacterial versus viral STIs. Poor hygiene after sex was also listed as cause of STIs in apparent confusion with genital tract infection. Other misconceptions – including that STIs were passed through shared toilets – were entangled with stigma.

#### Sources of information about STIs and genital health

As shown in Table 5, for those who had some information on STIs, schools were the most common source cited. Life Orientation (LO), part of the South African Department of Basic Education’s curriculum, is a compulsory school subject and includes comprehensive sexuality education (CSE) as a core component. Respondents described learning about STIs in LO classes from teachers, nurses, or through external programmes visiting their schools. Health care workers were also a common source of STI information. Despite health facilities being a main source of information about STIs, it was also stated that at clinics, the information provided mostly focuses on HIV, other STIs are rarely mentioned. For some respondents, the only discussions they had with anyone about STIs had been with nurse or other healthcare worker at the clinic or at mobile clinic visiting the school. Discussions with healthcare workers about STIs were mostly in the form of education and information. Some respondents had done self-research about STIs through speaking to a range of people and gathering opinions, and researching in books or on the internet.

**Table 5.** Illustrative quotations on Theme 2 – Sources of information about STIs and genital health.

Sources of information about STIs and genital health	
Learned at school	“we’ve discussed STIs at school” (WC, 15–17) “I was taught a lot about it (at school), I mean STIs, it’s an everyday topic… we about this talk about preventing these things… sicknesses that will infect you when you had sex without protection” (FS, 18–24) “I got information (on STIs) from school… nurses used to come to school and teach us about everything” (KZN, 18–24) “From the nurses who visited us at school… we learnt about it (STIs) at school during LO” (MPU, 18–24) “Oh! I know about that one (STIs)… in our LO classes… we normally do like maybe a speech on it…with posters… LO presentations” (WC, 18–24) “I learnt about it (STIs) at school in my Grade 7 class… I also learnt about it in Matric” (MPU, 18–24) “(I discussed STIs with) my high school matric English teacher… we would have general topics about such things” (GAU, 18–24) “We had a women’s… programme… those ladies would come to our school and they would speak about STI and also HIV” (WC, 18–24)
Self-research	“where I got the information (on STIs)… it was not from a programme but from another source… a book about health that I read as my hobby” (GAU, 18–24) “(I haven’t discussed STIs with anyone) I only read it on the Internet… I have no idea actually how it looks… but I used to search on the Internet” (EC, 18–24) “I got it (information on STIs) from internet, one of my friends was having a discharge that was not normal… We searched and found out it might be STI and then we did a search about STIs and causes” (MPU, 18–24)
Information from clinic/ health care providers	“I got it (information about STIs) from the clinic” (MPU, 18–24) “the doctors will always discuss it (STIs) with us and the nurses… they would make us aware” (WC, 18–24) “I have never spoke about it (STIs) with anyone that I know, but I have with a nurse at the clinic… She warned me about different kinds of STIs, I was told the importance of using a condom” (KZN, 18–24) “most times at the clinics they do not focus on telling you more about other diseases (STIs) all they know is about HIV… information around HIV, also billboards about it” (GAU, 18–24) “They also taught us about STIs at the mobile clinic at school, when you go for (HIV) testing, they will then talk about STIs” (KZN, 18–24)

#### Communication about STIs and genital health

Most qualitative respondents reported never having discussed STIs with anyone, consistent with quantitative findings. Many also said they had neither experienced an STI nor heard of others in their circles who had.

For those who had discussed STIs, as shown in Table 6, peers were a commonly listed source of discussion about STIs by respondents. However, the information received from peers was not always accurate, illustrating a lack of awareness about how to seek help or even that some STIs were treatable. Indeed, peers sometimes share partly accurate information, for example indicating some level of knowledge that male circumcision can reduce some STI risks but framing this in terms of blaming uncircumcised men having a sure STI infection. Some of those who had discussed STIs with friends described an initial internal stigma, but then realized with open communication experiences, that sharing information was useful. Being able to discuss STIs with peers was a facilitator to seeking help, and had the benefit of assuring AGYW that they were not alone, that other people also experienced STI symptoms and that they were treatable.

**Table 6.** Illustrative quotations on Theme 3 – Communication about STIs and genital health.

Communication about STIs and genital health	
Never discussed STIs	“Honestly, I have never heard of anyone who had that (STIs)” (FS, 18–24)
Teachers/sports coaches	“at netball we speak about such things (like STIs), we become open when we’re at netball… We laugh but Coach does tell us that in some things we shouldn’t laugh, these things happen in real life and you must take it seriously… we are open together and we give each other advice” (GAU, 18–24)
Peers	“we talked about it (STIs) with my girls” (MPU, 18–24)
Advice on health seeking from peers	“I have (discussed STI) with someone in my class… she knows a person that has it (STI) and that she does not know what to do about it, so I advised her to tell the person to go to the clinic for help” (GAU, 15–17) “There is someone I know that had an STI… the help I gave is to tell them to go to public clinic… I advised them to go to the clinic” (EC, 18–24)
Information from peers with STI experience	“I’ve known somebody that had it (STI) before… (they said) ‘I have so and so and it’s burning down there’” (WC, 15–17)
Inaccurate/incomplete information from peers	“(My friends speak about) a lot of bad things in terms of the vagina… they say there is no help according to them” (KZN, 18–24) “I don’t know much (about STIs)… I have heard my friends talk about it but I didn’t understand… When they explained (about STIs), they said something that happens to a girl who has multiple sex partners… I just keep quiet because they talk about a lot of things” (MPU, 18–24) “because of the foreskin that you have on your penis that is why you have those (diseases). Your foreskin has been holding… the bacteria this whole time” (GAU, 18–24)
Benefited from discussing STIs with peers	“My friend… She was happy to talk about it (STIs)… I did not feel good (discussing STIs with her)… but I ended up happy after realizing that it was health related” (FS, 18–24) “He told me his experience and then he said like there is itching and stuff like that… we really spoke about it because… I did not want to feel like I am the only one who had it… and then he told me ‘no like it is fine… (you can get) treatment and then it will be okay’” (WC, 18–24)
Communication with sexual partners	“I spoke to my boyfriend (about STIs)… he was surprised but engaging” (GAU, 18–24) “It was fine (discussing STIs with my boyfriend)… We talk, me and him… we advise each other as partners… I am open to him I do not hide anything” (GAU, 18–24)
Barriers to disclosing STI to partner	“I will tell him (if I had an STI)… but it would be difficult. He will not trust me, he will think of me as a bad girl” (MPU, 18–24)
Communication with female caregivers/family	“I was talking with my granny about it, and she was telling me that if I don’t use protection (condoms), I will get such diseases, she also told me that we’re not supposed to have sex before marriage” (FS, 15–17) “(I spoke to) my mum about STIs… She says it’s hygiene. She says if I wear a sanitary pad for a long time, obviously I’m going to get a reaction” (GAU, 18–24) “I discuss them (STIs) a lot with my sister… We discuss about these diseases… such as when someone has sex without protection… what kind disease they might have, you need to protect yourself every time… we talk about such things” (FS, 18–24) “I would tell my mother (if I had an STI) because she is the one I trust… and we talk a lot about health” (FS, 18–24) “(If I thought I had STIs) I would tell my mother, my sister and go to the clinic… Obviously I am going to freak out, open myself up to my mom and tell her, and then from there she will tell me what will do” (NW, 18–24)
Barriers to communicating about STIs with parents	“I feel like you cannot exactly tell a parent that I have a sexually transmitted disease they know we are (sexually) active but we should not be blunt with them about our sexual lives” (NW, 18–24)
STIs too embarrassing to disclose	“Being sexually active is embarrassing on its own, now imagine catching an infection… no, it’s embarrassing!” (GAU, 18–24)

A minority of the older qualitative respondents had discussed STIs with partners, generally describing open communication around sexual health as supportive and reciprocal. Several of the respondents who hadn’t experienced STIs themselves said they would disclose an STI to a sexual partner, framing this as a responsibility or as necessary to encourage partner testing. For others, disclosure to a partner was less straightforward; the fear of blame and accusations of infidelity made such conversations difficult.

Female family members were also mentioned as sources of STI knowledge, although advice was often framed in moralizing terms emphasizing sexual abstinence. Some said their mother was a trusted confidante and someone they could spoke to about genital health. Older sisters were also a source of information about STIs and protective behavior, and could provide valuable advice. While others admitted it would not be easy to confide in or disclosing an STI or vaginal infection to a parent, particularly given the sexual aspect of STIs. While some respondents suggested that advice from family members could help reduce panic and the anxiety of coping with an STI alone, others preferred not to disclose at all, citing embarrassment, shame, and anticipated judgment.

#### STI health care access and experiences

As illustrated by the quotations in Table 7, engagement with STI services among qualitative respondents was limited, though some recounted experiences of testing or treatment through schools and health facilities. Public health facilities were the most common point of care for those experiencing vaginal infections or STIs. When asked what they would do if they thought they had an STI, the majority of respondents said they would go to the clinic, which was widely described as the primary or only source of effective treatment. Some respondents asserted that seeking treatment for STIs is a straight forward process, and suggested that to effectively treat an STI, one needed to seek medical attention. Others stressed the importance of receiving treatment to prevent complications, taking medication as prescribed, and getting treated timeously so as to not cause worse complications, with some noting the dangers of leaving an STI untreated. Some of those respondents who shared their personal experiences with STIs recounted positive accounts of accessing STI health care, noting respectful treatment and supportive counseling.

**Table 7.** Illustrative quotations on Theme 4 – STI health care access and experiences.

STI health care access and experiences	
Hypothetical STI – would go to clinic	“(If I thought I had STIs) I will accept myself… and seek help… at the clinic… because that is where we would get help” (FS, 18–24) “(If I thought I had an STI) I’ll go to a clinic if it’s private or public… get tested and then get pills for it” (WC, 18–24) “(if I were to get an STI) I would go to the clinic… because in order to cure it, you need to go to the clinic” (MPU, 18–24) “(if I thought I had an STI) I would definitely go to the clinic. I know that it can lead to much more serious diseases and infections” (WC, 18–24) “(If I thought I had an STI) I’d go to the clinic to and check myself, and if I get medication, I will just drink it the way they tell me to. To protect myself from it spreading any further to the next stage” (NW, 18–24) “(If I thought I had STIs) I will go to the clinic and show them or tell them… that I think I have an STI… they test your urine… you show them your discharge, what’s wrong with it… they are the ones that prescribe with what you need to have and what sickness you have” (NW, 18–24)
Positive experience with STI services at clinic	“I did (receive help regarding STIs)… I got help from the clinic… I had a discharge and it was not stopping so I realized that this discharge was not normal… they did not shout at me and ask me why I did not use protection… They encouraged me to use protection in the future to avoid STIs” (KZN, 18–24) “I got it (treatment for STIs) from the clinic… they treated me well… they helped me” (MPU, 18–24)
Barriers to seeking care for STIs	“To be honest it takes courage… but if I see that I am ill and it’s beyond me, I do have to seek help… the main goal is to get help” (GAU, 18–24) “The first thing I would do is go to the clinic. If I feel something unusual down there…(but) I think they will shout at me because they always shout at people with STIs… (saying they) are irresponsible and reckless and they do not use protection” (KZN, 18–24) “At the clinic, they will not help me, they will only give me the pain-block and tell me that they do not have medication” (MPU, 18–24) “I went to the day hospital… they said I had an infection… they gave me the tablets… I would like something more private [laughs softly]… where you can feel more comfortable because… at the day hospital it is always full… there is one room where people come in and out… if I knew a place or someone to talk to that is more quiet or more private, then you can speak to them” (WC, 18–24)
Alternative health seeking behaviors after negative clinic experiences	“The health worker gave me pills that didn’t help… I kept on going there over and over again. The only help that I got that was from pharmacy when I explained… the pharmacist gave me thrush pills, which I took… I was better after that” (GAU, 18–24) “I did not receive treatment at the clinic… I was not getting any better, it was getting worse… when sitting on the chair the sores would be painful… (at the clinic) the doctor… gave me pills… (but) I was not getting healed… the pills they gave me did not help me with anything… I gave up… I then went to the Setswana health shop to buy medication… a traditional Setswana medicine… that’s where I really got helped… in three days the sores were healed… completely healed” (NW, 18–24)
Research and self-medication	“(If I had an STI) I will go to the internet and see what I can use… then I’ll go to the chemist, buy the tablets, then drink it” (EC, 18–24) “(If I thought I had an STI) I would Google a remedy if that do not work or go to the doctor to buy an antibiotic” (GAU, 15–17)
Wouldn’t know what to do	“(if I thought I had an STI)…[silent pause] I don’t actually know what I would do” (WC, 18–24) “(If I thought I had an STI) I really don’t know where or what I will do” (WC, 15–17)

In contrast to the positive experiences described above, other narratives related to feeling embarrassed and judged when seeking services for STIs. Despite many respondents emphasizing the necessity of seeking medical attention for STIs, it was acknowledged that it required courage to overcome the embarrassment of disclosing symptoms related to the genital region. Particularly given the common perception that those seeking treatment for an STI often get blamed and judged by healthcare providers. Respondents sharing their own experiences cited their concerns over the lack of confidentiality and privacy when accessing STI treatment at public health facilities.

Alternatives to public health care were also reported. Despite the strong orientation toward clinics, some respondents expressed mistrust in the public health system, saying they believed clinics were unhelpful, or that medication was not reliably available. For this group, private doctors or pharmacies were regarded as better alternatives, even if it meant paying for services. Some of those who had personal experiences with vaginal infections had turned to pharmacies when clinic treatment was perceived as ineffective. A few said they would rely on their own internet research and self-medicate using drugs purchased from a chemist. A less commonly cited option for those who expressed dissatisfaction with biomedical care was traditional medicine. Some respondents, particularly from the Cape Town site, admitted they would not know what to do if they suspected an STI.

## Discussion

The findings from this study illustrate knowledge, perceptions, and health-seeking behaviors related to STIs and vaginal infections among AGYW in South Africa. Fewer than one in five of the survey participants reported ever receiving an STI diagnosis from a healthcare provider, and only a small minority disclosed symptoms or treatment experiences in the past year. Qualitative findings revealed limited and often inaccurate knowledge about STIs and vaginal infections, with prevalent misconceptions and intersecting stigma negatively impacting AGYW’s willingness to disclose or seek help. Although most AGYW had not discussed STIs with anyone, those who had been able to discuss STIs with someone generally had benefited from the exchange, gaining information, assurance, and facilitating health seeking behavior. While some AGYW demonstrated accurate knowledge—particularly older respondents who more readily associated STIs with unprotected sex and multiple partners—others struggled to distinguish between symptoms. Most of the qualitative respondents stated that they would attend public clinics for STI care, but with notable accounts of avoidance due to anticipated judgment, embarrassment, or lack of confidentiality, particularly among younger participants. These findings illustrate a dual challenge: low rates of formal diagnosis and treatment uptake, coupled with gaps in STI-related knowledge and communication, both of which contribute to delayed or incomplete care-seeking for treatable STIs and vaginal infections among AGYW in South Africa.

### STI knowledge

Findings showed a wide range in STI knowledge among respondents. Although condomless sexual intercourse was identified as an STI transmission vector, notably no respondents mentioned oral or anal sex as possible STI transmission vectors, suggesting that this information should be included in education efforts. Some of the qualitative respondents were able to list symptoms such as discharge, malodor, sores, and itchiness, however there was also evidence of confusion and inaccurate information about STIs and vaginal infections. Confusion relating to symptoms of STIs and vaginal infections can be problematic due to the current South African national guidelines which follow a syndromic approach to the detection and treatment of STIs. The syndromic approach refers to the management of STIs being based on a patient presenting with symptoms and clinical signs associated with specific pathogens, and treatment given accordingly without running laboratory diagnostic tests, which can be costly and resource intensive (Kaida et al., 2018; Milford et al., 2025; Mullick et al., 2023). Given the high prevalence of asymptomatic STIs amongst adolescents in South Africa, the perception that vaginal discharge signals an STI risks incorrect syndromic treatment based on self-reported symptoms—leading to overtreatment of non-STI syndromes (e.g., bacterial vaginosis) and antimicrobial resistance (Kaida et al., 2018; Mullick et al., 2023). Syndromic management also means that asymptomatic STIs do not get identified or treated (Francis et al., 2018). Additionally, due to the fact that testing is limited to only a subset of STIs in the public health sector, identifying and treating STIs only focuses on a small set of STIs the clinics tests for and/or symptomatic STIs. Recommendations to address these challenges include shifting policy away from syndromic management of STIs toward innovative approaches such as real-time diagnostic testing and treatment provided in youth-friendly settings (Kaida et al., 2018).

### Sources of information about STIs

Although information about STIs is accessible at clinics, it was stated by respondents that the information provided mostly focuses on HIV, other STIs are rarely mentioned. One key source of information about STIs described by respondents was Life Orientation (LO) classes that are included in South African Department for Education’s national curriculum. Whilst the curriculum includes information on STIs, there are well documented challenges with the provision of LO classes, including the discomfort and reluctance of teachers (Munyai et al., 2023). Other sources of information on STIs respondents listed included discussions with peers, information received from parents/caregivers, information received at clinics, programmes or NGOs, and self-research. For some respondents, hearing peers speak about STIs could also be confusing and lead to misunderstandings as information was incomplete. Peer to peer knowledge sharing about SRH is often regarded as the preferred information source by adolescents and young people, however can lead to the dissemination of inaccurate information and misconceptions (Duby et al., 2022, 2025). Misconceptions and inaccurate information exacerbated stigma related to STIs. Self-research on the internet or in books was listed as one source of information by qualitative respondents. Some said that if they thought they had an STI they would use internet search engines to find out how to treat it themselves before seeking medical help. Evidence suggests that South African adolescents are increasingly turning to social media and digital platforms for health information (Shamu et al., 2020). Capitalizing on young people’s preference for doing self-research on topics that are seen as sensitive or embarrassing, such as STIs, mHealth interventions including platforms offering anonymous text message question and answer with trained counselors have been effective (Bergam et al., 2022).

### STI communication/disclosure

Most AGYW had never spoken to anyone about STIs, reflecting strong communication barriers. Among those who did, conversations were mainly with peers but also with partners, family, health workers, or trusted adults, and these exchanges generally provided information, reassurance, and support for seeking care. The ‘sexually transmitted’ nature of STIs, and their undeniable link to sexual intercourse, mean that talking about them necessitates an acknowledgement of sexual activity. In settings where communication around sex-related topics is bound by proscriptive socio-cultural guidelines that inhibit open communication, discussion of STIs is likely to be restricted (Duby et al., 2022). These socio-cultural proscriptions relating to sexuality discussions are compounded by the shame and stigma associated with STIs which further impede communication about STIs with partners and parents/caregivers (Scheinfeld, 2021). Shame and taboos related to the genital region, combined with STI related stigma, are also likely to pose barriers to adolescents’ disclosure of symptoms to health care providers (Morris et al., 2014). Evidence suggests that increased knowledge about STIs amongst adolescents and young people has the effect of reducing STI-related shame and stigma, and consequently increasing the likelihood of STI reporting and health seeking behaviors including STI testing (Dadzie et al., 2022; Scheinfeld, 2021). Evidence from the sub-Saharan African setting suggests that access to media messaging and education relating to STIs promotes utilization of STI screening and testing services (Dadzie et al., 2022).

### Healthcare service utilization

Findings showed that despite access barriers, medical facilities were the preferred treatment destination for STIs. Most qualitative respondents in this study said if they thought they had an STI they would go to the clinic. This was echoed in the survey findings, in which more than half of those respondents who had reported an STI related symptom in the past year had sought treatment from a clinic or hospital. Despite this relatively high accessing of medical treatment, some of the qualitative respondents articulated a lack of lack of trust in the public health care system saying it would be better to go to a private doctor. Respondents’ narratives illustrate the varied trajectories of young women seeking STI care, with issues of privacy, perceived effectiveness, and the quality of interpersonal interactions emerged as key factors shaping how and where young women accessed STI services. While some experienced respectful and effective treatment at public clinics, others encountered barriers that prompted them to seek help through pharmacies or traditional healers. Illustrating medical pluralism in the South African setting, some of the qualitative respondents described using traditional medicines and remedies to try and treat STIs, citing a greater level of trust in traditional health care practitioners and formulations than the public health care sector and biomedical/pharmaceutical treatments. Reporting of seeking treatment from a traditional healer among survey respondents was relatively low, albeit with slightly higher use of traditional healers amongst those AGYW under 18 years. Given this context of medical pluralism in South Africa and the high levels of public trust in the traditional medical sector, further research is warranted into how STIs and genital infections are conceptualized and how these representations influence health seeking behavior (Meyer-Weitz et al., 1998; Mulaudzi & Makhubela-Nkondo, 2006).

### STI stigma: STIs as shameful/embarrassing

Among those survey participants who had reported an STI related symptom in the past year, almost a third of those AGYW under 18 years reported that they did not get any treatment for their STI symptoms. Among qualitative respondents, many echoed this, saying that they would be too ashamed and embarrassed to seek any help if they thought they had an STI. Respondents articulated the perception that those seeking treatment for an STI often get blamed and judged by healthcare providers. Evidence from prior research in South Africa and elsewhere suggests that even when young people are aware of STI services available, there are several barriers to using these services; one of the main barriers being fear of judgment and scolding by health care workers (Mokgatle et al., 2021; Sales et al., 2007). Respondents admitted that seeking medical help for a suspected STI took courage to overcome embarrassment and shame. Although it was suggested that seeking help from a health care professional would be less embarrassing than disclosing to someone else in family or social networks. The embarrassment and barriers to openly communicate about STIs is also linked with the sociocultural restrictions related to discussing the genital region without using euphemistic terms (Duby et al., 2022). The reluctance to disclose STIs due to shame and embarrassment impedes AGYW’s ability to seek help. Prior literature describes the shame and stigma associated with STIs, and the impact these have on delaying STI healthcare and impeding communication (Morris et al., 2014; Cunningham et al., 2009). However, to the authors’ knowledge, there is no existing data on STI shame and stigma amongst adolescent girls and young women in the South African context.

### Genital shame

The shame and embarrassment associated with STIs articulated by respondents had partly to do with the fact that they affect the genital region. Literature suggests that STIs carry a specific weight of shame, stigma and social judgment (Sales et al., 2007; Scheinfeld, 2021). Part of this shame and stigma relates to sociocultural perceptions of the genital area, more specifically the vagina and its secretions, inherently considered shameful and associated with disgust (Duby et al., 2020). However whilst some of the shame and embarrassment of STIs is due to their relationship to the genital area, STIs are socially perceived as more shameful that other genital/gynaecological conditions (Cook, 2014). Perceptions of vaginal infections and STIs as dirty, associated with bad smells and unsightly sores, add to stigma and shame, and set them apart from other illnesses (Shefer et al., 2002). The fact that STIs and vaginal infections such as BV may manifest in discolored or malodourous discharge heighten the preexisting disgust around any secretions from the vagina (Duby et al., 2020). Additionally, some South African languages equate discharge with conceptions of ‘dirt’ and uncleanliness (Mulaudzi & Makhubela-Nkondo, 2006).

### Adolescent sexuality taboos

The shame and embarrassment associated with STIs was also related to the fact that they are transmitted sexually. The fear of judgment expressed by qualitative respondents in our study highlights what has been described as a powerful moral component in clinical situations, specifically STI testing, where positive diagnoses may carry assumptions about behavior and lay blame on the deviant and irresponsible individual (Cook, 2014; Morris et al., 2014; Shefer et al., 2002). The moralistic judgements related to STIs are further compounded by sociocultural framing suggesting that adolescent sexuality should be repressed and controlled (Duby et al., 2022). Evidence from South Africa and other sub-Saharan African countries corroborates the finding that young people often feel concerned about health care providers scolding them for being sexually active, and making assumptions that they are promiscuous if they require STI treatment (Avuvika et al., 2017; Mokgatle et al., 2021). Internalized shame and blame relating to SRH issues, whether STIs, early pregnancy, or HIV – tend to result in a reluctance to seek care and impeded ability to access services and adopt preventative behaviors (Duby et al., 2025; Cunningham et al., 2009). Although shame can have the negative impact of impeding health seeking behaviors and delaying access to treatment, it has been argued that shame related to STIs can also be a positive motivating factor for condom use by adolescents who want to avoid contracting ‘shameful’ STIs (Sales et al., 2007; Cunningham et al., 2009). Despite barriers, most participants indicated they would seek clinic help if they suspected an STI, demonstrating a prioritization of health over embarrassment; this could be leveraged in messaging around STIs for AGYW.

## Limitations

Firstly, the qualitative interview questions did not delineate between vaginal infections such as thrush and other STIs. Secondly, analysis of interview data was performed on English transcripts, therefore the nuances of terminology relating to STIs and vaginal infections in the original languages that interviews were conducted in were not captured. Thirdly, it is likely that the social desirability bias related to self-reports of STI symptoms and experiences were enhanced due to the stigmatized and shameful nature of STIs and the embarrassment of discussing the genital region. This is illustrated by the fact that almost a quarter of survey respondents preferred not to answer the question about STI symptoms. Lastly, since most STIs are asymptomatic in women (Francis et al., 2018), and the survey relied on self-reports which are likely to be lower end estimates. Therefore, many AGYW with STIs would not be identified because they were asymptomatic.

## Conclusions and implications

South African AGYW are disproportionately affected by STIs, as compared to the general population, and to males of the same age due to complex and multifaceted, biological and behavioral factors interacting with social determinants of health, gender inequities, and stigma related to STIs (Dadzie et al., 2022; Dookhith, 2022; Harryparsad et al., 2023; Jarolimova et al., 2023; Onywera et al., 2022; Peters et al., 2022). The findings from our study illustrate the pervasive stigma and shame associated with STIs, which impede AGYW accessing timeous and appropriate services and treatment. Addressing STI stigma may be achieved through the provision of integrated SRH services, inclusive of pregnancy testing, prevention and care inclusive of contraceptive provision and antenatal services; HIV testing, prevention and treatment inclusive of PrEP and antiretroviral HIV treatment provision; STI testing, prevention and treatment; alongside a range of holistic services such as counseling, psychosocial support and education (Morris et al., 2014). Such integration may have multi-fold effects of reducing not only STI stigma, but also HIV stigma, PrEP stigma, and reduce barriers to accessing any one of the included services.

Given the multiple barriers to consistent condom use among adolescents and young people in South Africa, and the potential that with increased targeting of PrEP provision to AGYW, risk compensation further reduces motivation to use condoms, there is an urgent need to ensure that treatable STIs remain on the agenda (Duby et al., 2021; Joseph Davey et al., 2025). Reduced condom use could increase the prevalence of STIs among adolescents and young people in South Africa in turn increasing AGYW’s risk of HIV infection despite the protective effect of PrEP for those taking PrEP. Thus, programmes need to encourage condom use among all PrEP users whilst also increasing STI testing and treatment. Promoting condom use in the context of increasing uptake of PrEP is important because PrEP does not prevent against the acquisition of STIs.

This study highlights the urgent need to address gaps in STI knowledge and barriers to communication and health service utilization among AGYW in South Africa. The findings demonstrate how pervasive stigma and shame related to STIs impede timely health-seeking for treatable infections. Strengthening comprehensive, integrated youth-friendly SRH services that prioritize confidentiality, ensure non-judgmental care, and expand beyond an HIV-centric focus toward inclusive STI education, testing, and treatment is essential. Tailored interventions that leverage trusted communication channels—such as schools, peers, digital platforms, and family networks—could play a critical role in normalizing STI dialogue, reducing stigma and shame, and promoting preventative behaviors. Importantly, the balance between increasing PrEP uptake and safeguarding consistent condom use must be emphasized to mitigate rising STI prevalence and its compounding effect on HIV vulnerability. By framing STI services within comprehensive adolescent-friendly health systems, South Africa can move toward more effective prevention, timely diagnosis, and equitable access to STI treatment that empowers AGYW to take charge of their sexual and reproductive health.

## Supplementary Material

Supplemental Material
