# Supplementary material for: “It’s burning down there”: A Mixed-Methods Study on Sexually Transmitted and Other Vaginal Infections Among Adolescent Girls and Young Women in South Africa
Source: Int J Sex Health. 2026 Mar 12;38(3):1174–89. doi: 10.1080/19317611.2026.2637641 (PMC13531938; doi:10.1080/19317611.2026.2637641)
Supplement: Supplementary file 1 [file WIJS_A_2637641_SM3016.docx]

**Supplementary Information**

**Survey questions on STIs**

The data presented in this paper derives from the following survey questions pertaining to Sexually Transmitted Infections (STIs):

- *Did a doctor or nurse ever say you had a sexually transmitted infection (STI) in or around your private parts (vagina, anus/bum)?*
- *In the past year, did you experience any of the following on your private areas (vagina, anus/butt)?: itching, lumps, warts, rash, redness, smelly discharge, pain or burning upon urination.*
- *If you experienced any one of these on your private parts in the past year, where did you go to get treatment?*

**Qualitative interview questions on STIs**

The qualitative data presented in this paper are derived from the following interview questions pertaining to Sexually Transmitted Infections (STIs):

- *Have you ever discussed STIs with anyone?*
- *Could you tell us about any experiences you’ve had with STIs, or anyone you know.*
- *Have you ever received any services for STIs?*
  - ***If Yes:*** *Where did you receive these services from?*
    - *What made you choose to access the services from this place?*
    - *How did you feel when you were accessing the services?*
    - *How easy was it to receive these services?*
    - *Were there any services you would have liked to access but were not able to?*

*Why were you not able to?*

*What would make it easier to access these services?*

- *If you thought you had an STI, what would you do?*
